# Supplementary material for: GLW7.1, a Strong Functional Allele of Ghd7, Enhances Grain Size in Rice
Source: Int J Mol Sci. 2022 Aug 5;23(15):8715. doi: 10.3390/ijms23158715 (PMC9369204; doi:10.3390/ijms23158715)
Supplement: Supplementary file 1 [file ijms-23-08715-s001.zip › Supplementary figures of GLW7.1 .pdf]

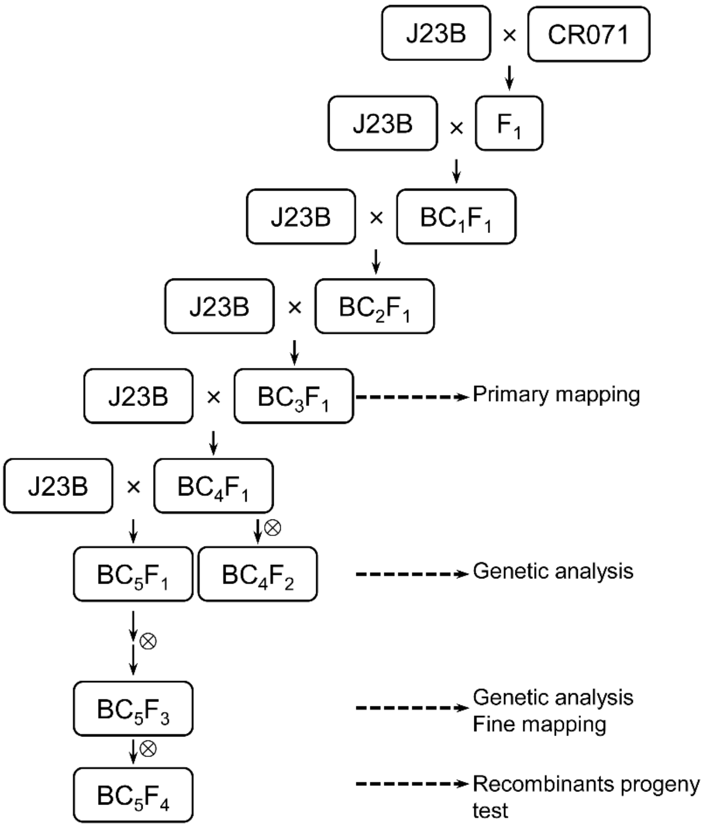


**Figure S1.** Pedigree chart of experimental materials.

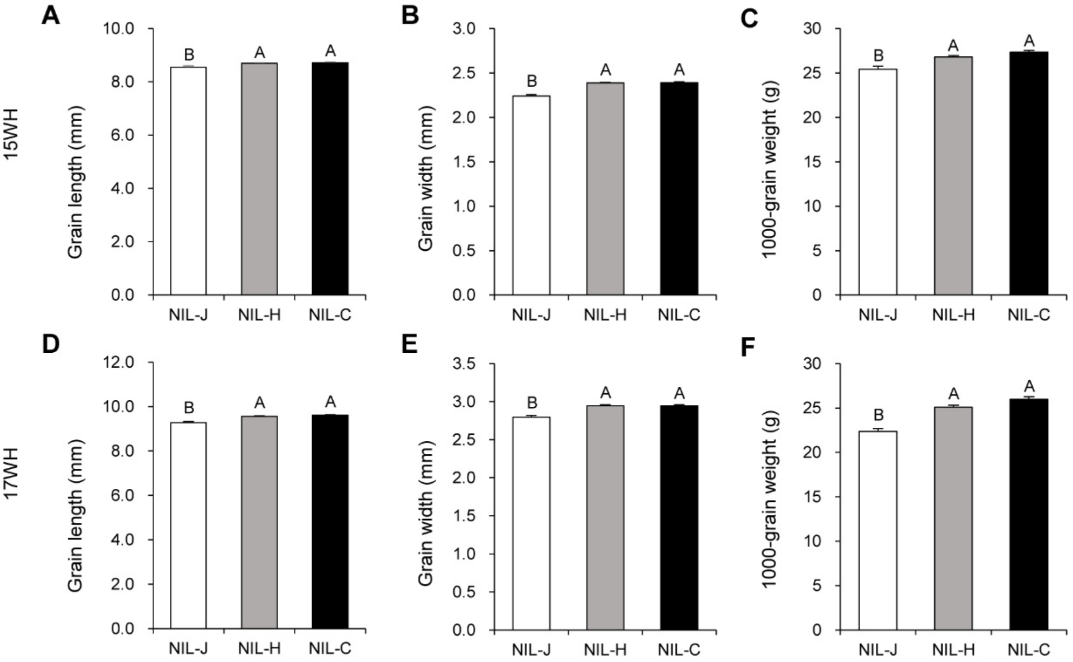


**Figure S2.** Genetic effect of *GLW7.1* on grain size in two NIL populations.

**(A)** Grain length, **(B)** Grain width, **(C)** 1000-grain weight during 2015 using a NIL population of 130 individuals. **(D)** Grain length, (**E)** Grain width, **(F)** 1000-grain weight during 2017 using a NIL population of 70 individuals. NIL-J, NIL-H and NIL-C represent for NIL plants carrying homozygous J23B allele *glw7.1*, heterozygous allele *GLW7.1*/*glw7.1* and homozygous CR071 allele *GLW7.1,* respectively. All phenotypic data in **(A–F)** were measured from paddy-grown NIL plants grown under normal cultivation conditions in Wuhan. Data were represented as mean ± s.e.m. and Duncan's multiple range tests were used to conduct statistical analysis (A, B and C indicate $P < 0.01$).

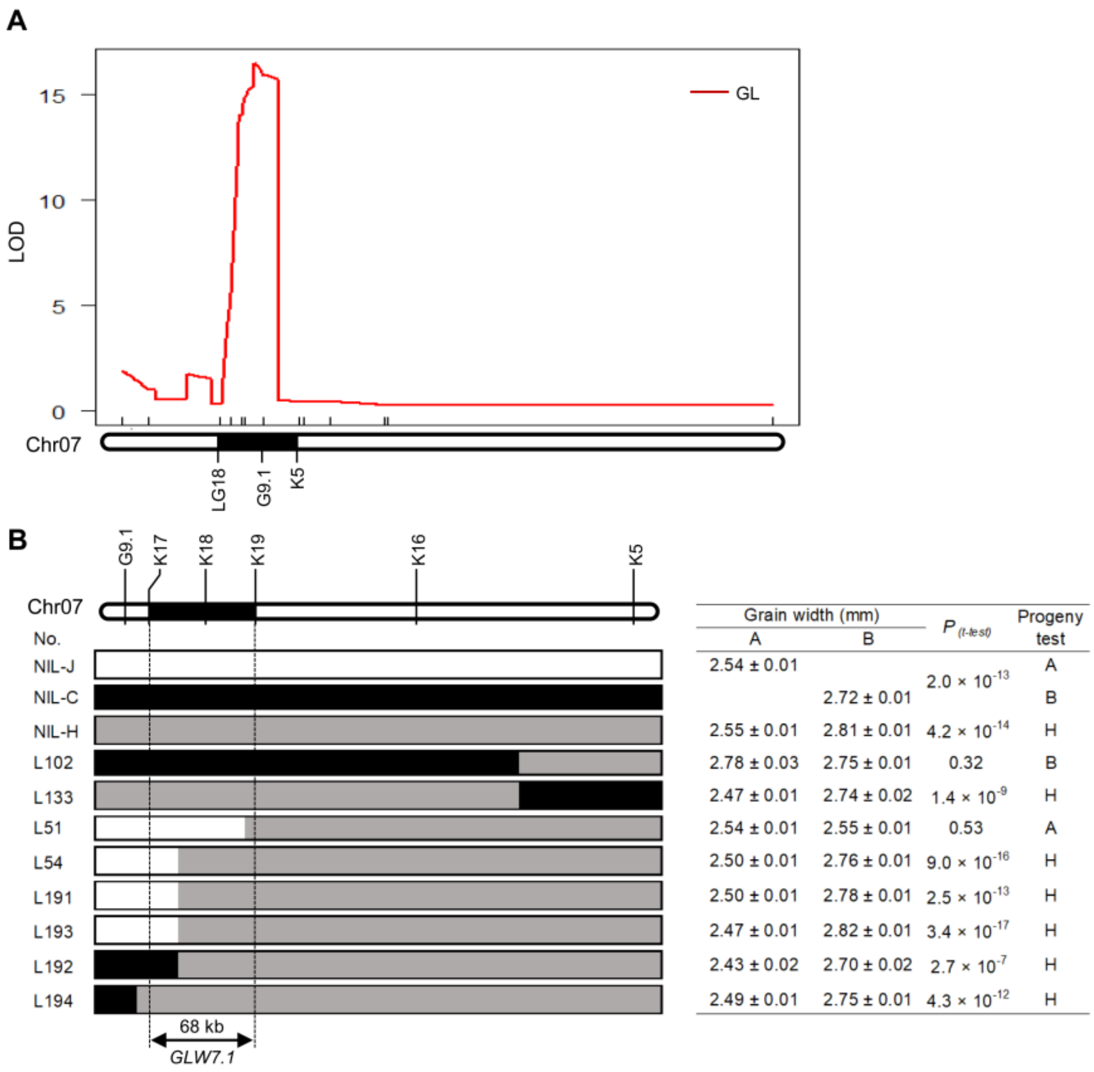


| No. | Grain width (mm) A | Grain width (mm) B | $P_{(t\text{-}test)}$ | Progeny test |
|---|---|---|---|---|
| NIL-J | 2.54 ± 0.01 | | $2.0 \times 10^{-13}$ | A |
| NIL-C | | 2.72 ± 0.01 | | B |
| NIL-H | 2.55 ± 0.01 | 2.81 ± 0.01 | $4.2 \times 10^{-14}$ | H |
| L102 | 2.78 ± 0.03 | 2.75 ± 0.01 | 0.32 | B |
| L133 | 2.47 ± 0.01 | 2.74 ± 0.02 | $1.4 \times 10^{-9}$ | H |
| L51 | 2.54 ± 0.01 | 2.55 ± 0.01 | 0.53 | A |
| L54 | 2.50 ± 0.01 | 2.76 ± 0.01 | $9.0 \times 10^{-16}$ | H |
| L191 | 2.50 ± 0.01 | 2.78 ± 0.01 | $2.5 \times 10^{-13}$ | H |
| L193 | 2.47 ± 0.01 | 2.82 ± 0.01 | $3.4 \times 10^{-17}$ | H |
| L192 | 2.43 ± 0.02 | 2.70 ± 0.02 | $2.7 \times 10^{-7}$ | H |
| L194 | 2.49 ± 0.01 | 2.75 ± 0.01 | $4.3 \times 10^{-12}$ | H |

**Figure S3.** Map-based cloning of *GLW7.1*.

**(A)** Fine mapping of the *GLW7.1* locus using phenotype of 600 recombinants and 70 non-recombinants derived from 30,000 $BC_5F_2$ segregants. **(B)** Genotypes and phenotypes of the recombinants. Grain width (mean ± s.e.m.) of three near-isogenic lines (NILs), and recombinant lines (L102, L133, L51, L54, L191, L193, L192, L194). White bars represent chromosomal segments for J23B homozygote (progeny test named as A), black for CR071 homozygote (progeny test named as B), and grey for heterozygotes (progeny test named as H). Homozygous progenies from each line were harvested to compare phenotypic differences. The student's *t*-test was used to produce *P* values.

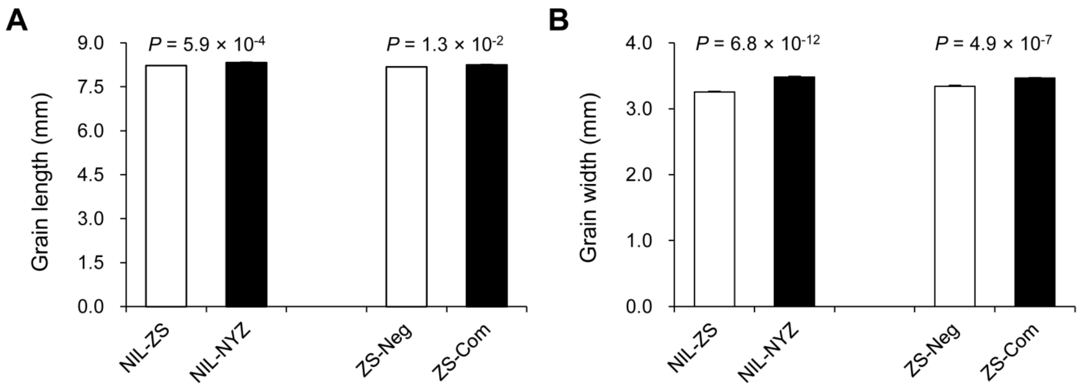


**Figure S4.** Effects of the other two *Ghd7* alleles (*Ghd7-1* and *Ghd7-2*) on grain size.

**(A)** Grain length. **(B)** Grain width. NIL-ZS, the NIL line without *Ghd7* fragment in the Zhenshan97 (*Ghd7-0*) background. NIL-NYZ, the NIL line with *Ghd7-2* allele fragment which was derived from a tropical *japonica* variety Nanyangzhan. ZS-Neg, the negative complementary line in the Zhenshan97 background. ZS-Com, the complemental line expressing Minghui63 (*Ghd7-1*) allele. All phenotypic data were measured from paddy-grown plants grown under normal cultivation conditions. Data were represented as mean ± s.e.m. ($n$ = 10). The student's $t$-test was used to produce $P$ values.

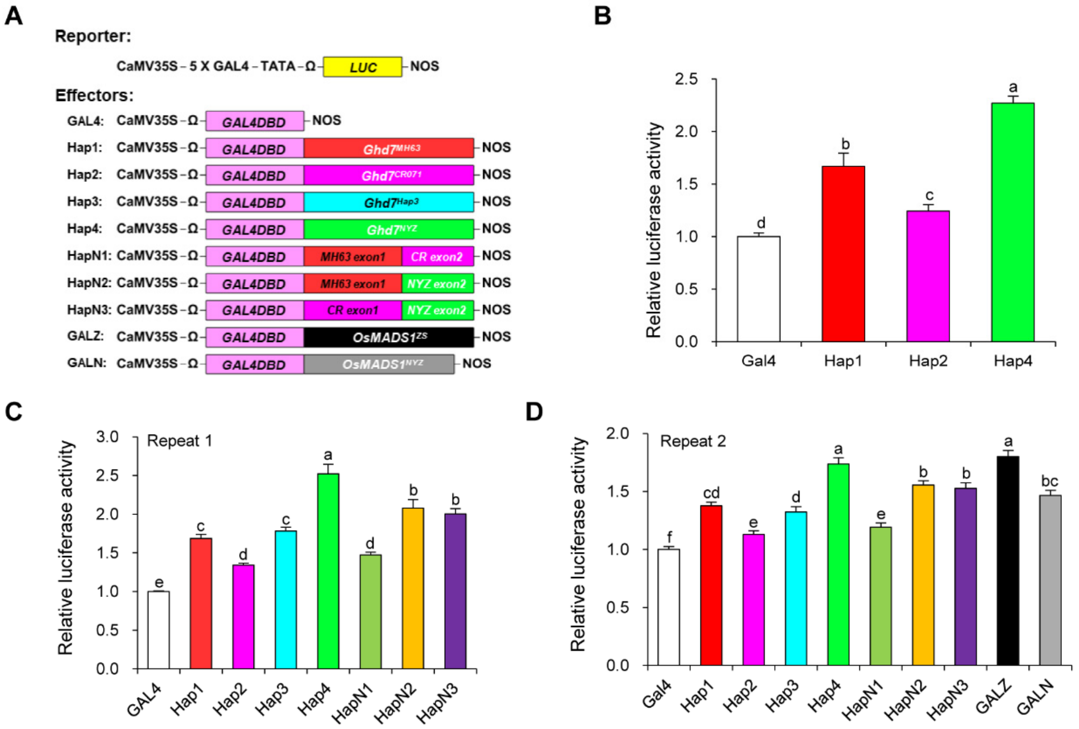


**Figure S5.** The transactivation activity of different allelic GHD7 proteins.

**(A)** Seven allelic GHD7 were fused to the GAL4 DNA-binding domain (GAL4DBD). The relative activity of firefly luciferase (LUC) under control of the 5×GAL4-binding element was measured. Renilla luciferase (REN) activity was used as internal control. The two allelic OsMADS1 ($OsMADS1^{ZS}$ and $OsMADS1^{NYZ}$) were used as positive control. **(B)** The transactivation activity of Hap1, Hap2 and Hap4 ($n$ = 10). **(C)** Repeat 1 of the transactivation activity of seven allelic GHD7 ($n$ = 11). **(D)** Repeat 2 of the transactivation activity of seven allelic GHD7 ($n$ = 10). Data were represented as mean ± s.e.m. and Duncan's multiple range tests were used to conduct statistical analysis (a, b, c, d ,e and f indicate $P < 0.05$).

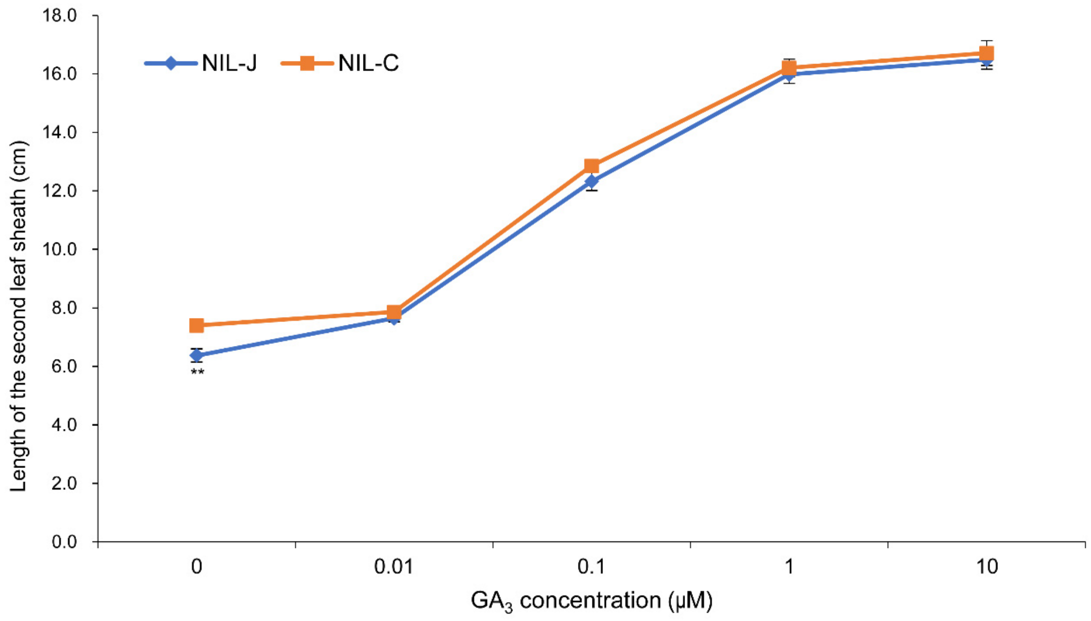


**Figure S6.** Elongation of the second leaf sheath in NIL-J and NIL-C in response to $GA_3$. The germinated seeds were grown in the nutrient solution that contained various concentrations of $GA_3$ and incubated at 28 °C under 13-h light/11-h dark conditions. After 10 days, the length of the second leaf sheaths was measured. Data were represented as mean ± s.e.m. ($n \geq 15$). Student's *t*-test was used to produce *P* values (** indicates $P < 0.01$).

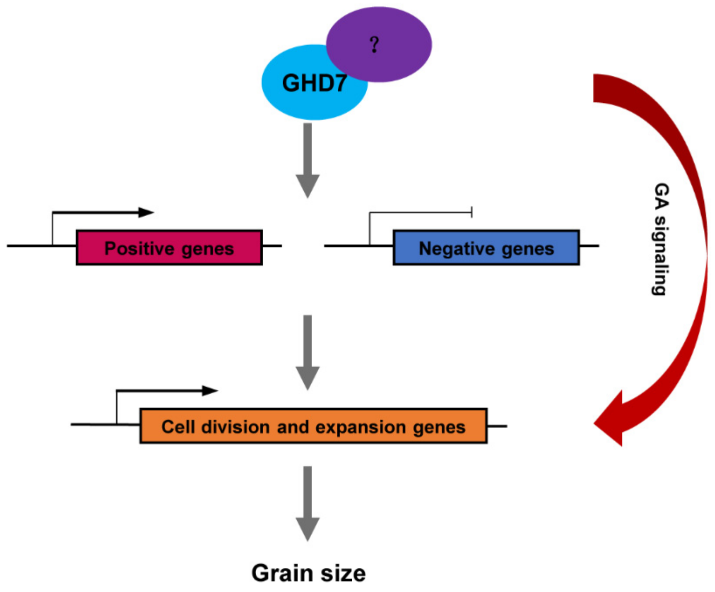


**Figure S7.** A hypothetical model showing that the GHD7 modulates rice grain size by promoting cell division and expansion. GHD7 interacts with several grain size related proteins, up-regulating the positive regulation genes of grain size and down-regulating the negative,

thereby promoting the transcription of downstream cell division and expansion genes. Additionally, GHD7 could also participate in the GA biosynthesis to increase endogenous GA level, and then stimulate downstream cell division and expansion genes expression in response to GA. Arrow-bars indicate the promotion of gene expression and T-bar indicates the inhibition of gene expression.

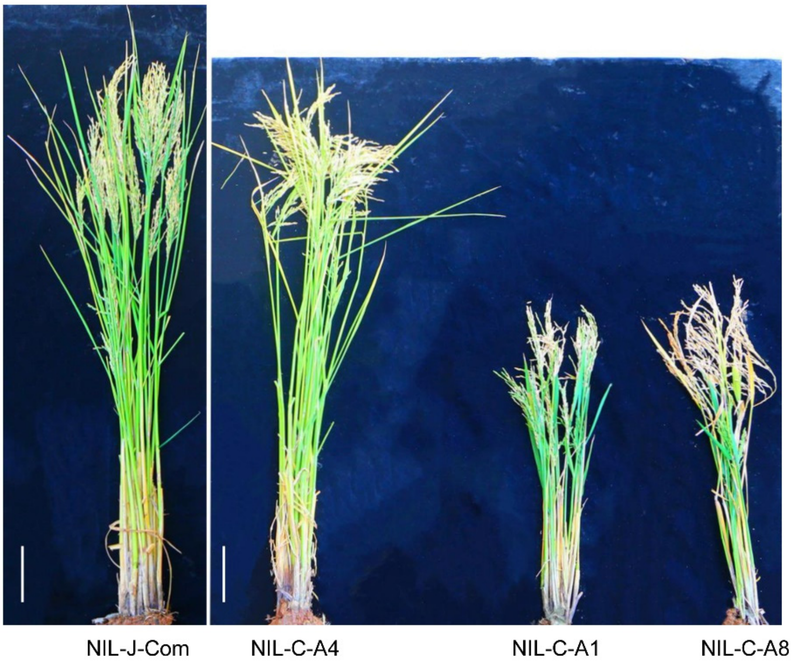


**Figure S8.** The gross morphology of transgenic plants. Scale bar: 10 cm.
